# Post-mortem assessment in vascular dementia: advances and aspirations

**DOI:** 10.1186/s12916-016-0676-5

**Published:** 2016-08-26

**Authors:** Kirsty E. McAleese, Irina Alafuzoff, Andreas Charidimou, Jacques De Reuck, Lea T. Grinberg, Atticus H. Hainsworth, Tibor Hortobagyi, Paul Ince, Kurt Jellinger, Jing Gao, Raj N. Kalaria, Gabor G. Kovacs, Enikö Kövari, Seth Love, Mara Popovic, Olivia Skrobot, Ricardo Taipa, Dietmar R. Thal, David Werring, Stephen B. Wharton, Johannes Attems

**Affiliations:** 1 Institute of Neuroscience, Newcastle University, Newcastle upon Tyne, UK; 2 Department of Immunology, Genetics and Pathology, Uppsala University, Uppsala, Sweden; 3 Hemorrhagic Stroke Research Program, Department of Neurology, Massachusetts General Hospital Stroke Research Center, Harvard Medical School, Boston, MA USA; 4 Stroke Research Team, University Hospital Lille, Lille, France; 5 Departments of neurology and Pathology, University of California, San Francisco, USA; 6 Department of Pathology – LIM-22, University of Sao Paulo Medical School, São Paulo, Brazil; 7 Institute of Cardiovascular and Cell Sciences, St George’s University of London, London, UK; 8 Department of Neuropathology, University of Debrecen, Debrecen, Hungary; 9 Sheffield Institute for Translational Neuroscience, Sheffield, UK; 10 Institute of Clinical Neurobiology, Vienna, Austria; 11 Neurological Department, Peking Union Medical College Hospital, Beijing, China; 12 Institute of Neurology, Medical University of Vienna, Vienna, Austria; 13 Department of Mental Health and Psychiatry, University of Geneva, Geneva, Switzerland; 14 Clincial Neurosciences, University of Bristol, Bristol, UK; 15 Institute of Pathology, Faculty of Medicine, University of Ljubljana, Ljubljana, Slovenia; 16 Unit of Neuropathology, Centro Hospitalar do Porto, University of Porto, Porto, Portugal; 17 Department of Neuroscience, KU-Leuven and Department of Pathology, UZ-Leuven, Leuven, Belgium; 18 Institute of Neurology, University College London, London, UK

**Keywords:** Vascular dementia, Vascular cognitive impairment, Cerebrovascular disease, Cerebrovascular lesions, Neuropathology, Magnetic resonance imaging, Post-mortem MRI, Mixed dementia

## Abstract

**Background:**

Cerebrovascular lesions are a frequent finding in the elderly population. However, the impact of these lesions on cognitive performance, the prevalence of vascular dementia, and the pathophysiology behind characteristic in vivo imaging findings are subject to controversy. Moreover, there are no standardised criteria for the neuropathological assessment of cerebrovascular disease or its related lesions in human post-mortem brains, and conventional histological techniques may indeed be insufficient to fully reflect the consequences of cerebrovascular disease.

**Discussion:**

Here, we review and discuss both the neuropathological and in vivo imaging characteristics of cerebrovascular disease, prevalence rates of vascular dementia, and clinico-pathological correlations. We also discuss the frequent comorbidity of cerebrovascular pathology and Alzheimer’s disease pathology, as well as the difficult and controversial issue of clinically differentiating between Alzheimer’s disease, vascular dementia and mixed Alzheimer’s disease/vascular dementia. Finally, we consider additional novel approaches to complement and enhance current post-mortem assessment of cerebral human tissue.

**Conclusion:**

Elucidation of the pathophysiology of cerebrovascular disease, clarification of characteristic findings of in vivo imaging and knowledge about the impact of combined pathologies are needed to improve the diagnostic accuracy of clinical diagnoses.

## Background

Cerebrovascular disease (CVD) is highly prevalent in brains of the elderly. However, its impact on cognition is less clear and while prevalence rates of vascular dementia (VaD) are high in clinical studies CVD is rarely found to be the neuropathological correlate of clinical dementia in post-mortem studies. In this review we highlight some of the current problems in the diagnosis of CVD and present novel approaches that may prove helpful to elucidate the impact of CVD on cognitive performance.

## Methods

This article was conceived at the 9^th^ International Congress of Vascular Dementia by participants of the Neuropathology symposium following a discussion on current problems regarding the clinical and pathological diagnosis of VaD and CVD.

## Neuropathology of cerebrovascular disease

### Degenerative cerebral vessel pathology

Three diseases of cerebral blood vessels mainly contribute to vascular cognitive impairment (VCI) and/or VaD: (1) atherosclerosis (AS), (2) small vessel disease (SVD) and (3) cerebral amyloid angiopathy (CAA). AS is a degenerative vessel disorder affecting large to medium sized cerebral arteries, most commonly the basilar artery and the circle of Willis [1], and results in the formation of atherosclerotic plaques due to accumulation of cholesterol-laden macrophages. Mature atherosclerotic plaques calcify, which may lead to narrowing of the artery lumen, and they are prone to rupture, resulting in subsequent thrombosis and potential thromboembolism [2].

SVD encompasses three degenerative alterations of the vessel walls of smaller cerebral arteries and arterioles. The first, SVD-AS, has a similar pathogenesis to large vessel AS but affects small intracerebral and leptomeningeal arteries (200–800 μm in diameter), which develop microatheromas. The second, lipohyalinosis, affects smaller arteries and arterioles (40–300 μm in diameter) and is characterised by asymmetric fibrosis/hyalinosis associated with cholesterol-laden macrophage infiltration that can occur with or without plasma protein leakage as a result of blood–brain barrier (BBB) breakdown. The third, arteriolosclerosis, presents as concentric hyaline thickening of small arterioles (40–150 μm) that may lead to stenosis of the blood vessel [3]. SVD initially manifests as lipohyalinosis and arteriolosclerosis in vessels of the basal ganglia, that is, the putamen and globus pallidus, and then in leptomeningeal arteries. By contrast, SVD-AS develops in the leptomeningeal arteries, and affects brain stem arterioles only in the end stages of SVD. Cortical vessels on the other hand remain relatively free of SVD pathology [4].

CAA is characterised by the deposition of amyloid-beta (Aβ) (predominately Aβ-40) in the vessel walls of leptomeningeal and cortical arteries, arterioles, capillaries and, rarely, veins [5]. This results in the loss of smooth muscle cells, disruption of vessel architecture and, in very severe stages, Aβ depositions in the adjacent neuropil (i.e. dyshoric changes). Topographically, CAA usually presents in the neocortex, with more frequent and severe deposition seen in the occipital region, followed by the allocortex and cerebellum, and finally in the basal ganglia, thalamus and white matter [6].

### Cerebrovascular lesions

**Fig. 1:**
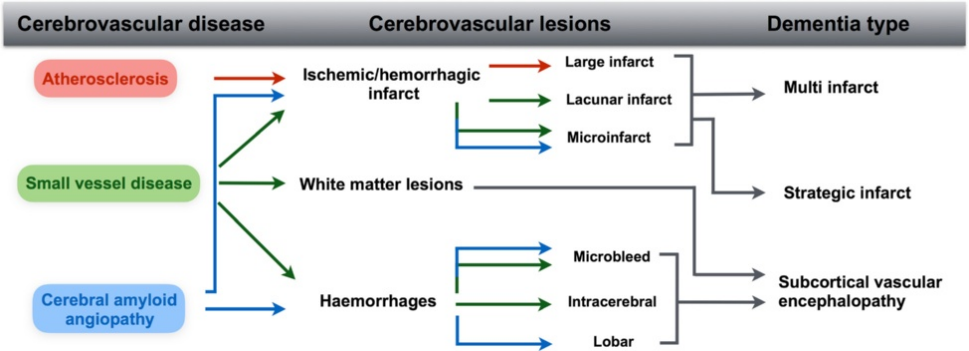
Schematic diagram illustrating the three most commonly observed cerebrovascular diseases and their resulting cerebrovascular lesions that may lead to specific types of vascular dementia

AS, SVD and CAA can all lead to various cerebrovascular lesions (CVLs), including infarcts, haemorrhages and white matter lesions (WMLs). Ischaemic infarcts are typically observed after thrombotic or thromboembolic occlusion of large to medium arteries, often as the result of an AS plaque rupture. Haemorrhagic infarcts can occur in infarcted regions in which the remaining vessels have fragile vessel walls as a result of SVD or CAA, or they may be caused by venous obstruction; less commonly, haemorrhagic infarcts in the brain can be caused by collateral blood influx into an infarcted area [7]. Large infarcts (>15 mm^3^) are frequently the result of thrombotic (AS) or thromboembolic (AS, extracranial AS, cardiogenic) occlusion of the vessel lumen [8]. Lacunar infarcts, that is, cavitating infarcts (5–15 mm^3^), are largely confined to the white matter and subcortical grey matter, and they are therefore primarily associated with SVD [9]. Microinfarcts (<5 mm in diameter) can be present in both the cortex and white matter, and they are associated with CAA and SVD respectively [3]. While cerebral haemorrhages (>10 mm in diameter) can result from all types of vessel disorders, those located in the subcortical grey matter, brain stem and deep white matter are strongly associated with SVD, whereas lobar haemorrhages are most commonly associated with CAA. Small haemorrhages (<10 mm in diameter) and microbleeds may histologically appear as extravasations of erythrocytes, but more frequently the only histological correlates of microbleeds diagnosed by in vivo imaging are haemosiderin-laden macrophages in the perivascular space, which may or may not be the residue of a bleed. In the cortex, small haemorrhages and microbleeds are associated with CAA [10], whereas those located in the white matter, subcortical grey matter and brain stem are associated with SVD [11]. WMLs encompass structural damage histologically characterised by white matter rarefaction, that is, demyelination and axonal loss, mild astrocytosis, oedema and macrophage reaction [3]. Of note, subcortical U-fibres are usually spared. WMLs are generally assumed by clinicians and radiologists to be the result of SVD-related chronic hypoperfusion and BBB alterations [12–14], although it is unclear if periventricular WMLs and deep WMLs share the same pathogenesis (Fig. 1). In addition, severe neurodegenerative pathology in the cortex has recently been suggested to cause WMLs (see section ‘White matter hyperintensities’).

### Pathological classifications of vascular dementia

CVLs can result in ‘pure’ VaD, that is, extensive vascular lesions, without widespread neurodegenerative pathology such as Alzheimer’s disease (AD) or Lewy body pathology, which explains the clinical dementia. VaD can be classified into three major forms depending on lesion distribution: multi-infarct dementia, strategic infarct dementia or subcortical vascular encephalopathy. Multi-infarct dementia is characterised by multiple lacunar infarcts and microinfarcts, as well as small and/or large infarcts in the cortex and subcortical regions. The total amount of damaged cerebral tissue results in a significant decrease in functional brain capacity, surpassing the threshold for cognitive impairment. In contrast, strategic infarct dementia is the result of a single infarct in a strategic region of the brain that results in significant cognitive deficits, for example, a single lacunar or microinfarct in the hippocampus can lead to marked memory impairment [15, 16]. Lastly, subcortical vascular encephalopathy (synonymous with Binswanger’s disease) describes confluent severe demyelination and axonal loss in the white matter with sparing of subcortical U-fibres ([13, 15, 16]; for review see [17]).

### Comorbidity of cerebrovascular disease and Alzheimer’s disease pathology

A large proportion of patients with dementia who have significant CVLs also exhibit more severe concomitant AD pathology [18], such as deposits of hyperphosphorylated tau (HPτ) and Aβ, and thus fulfil the neuropathological criteria for AD (Braak neurofibrillary tangle [NFT] stage V/VI, Consortium to Establish a Registry for Alzheimer’s Disease [CERAD] score C and Aβ phase 5 according to the National Institute on Aging–Alzheimer’s Association [NIA–AA] guidelines [19–22]). They are therefore classified as having mixed AD/VaD. The distinction between AD, VaD and mixed AD/VaD remains controversial and poses a difficult challenge (see section ‘Clinico-pathological correlations and mismatch in VaD and mixed VaD/AD’).

## Prevalence of vascular dementia

In clinical population-based series, the prevalence of VaD/VCI averages 8–15.8 % (in Japan, 23.6–35 %) with standardised incidence rates between 0.42 and 2.68 per 1000/year, increasing with age [23]. The range is broader in clinical studies using convenience series from western memory clinics, varying from 4.5 to 39 % [23]. However, the prevalence rates of VaD/VCI are unlikely to be accurate in any of these series because even the best clinical diagnostic criteria show only moderate sensitivity (approximately 50 %) and variable specificity (range 64–98 %) [23, 24]. VaD in autopsy series also varies tremendously, ranging from 0.03 to 58 % [23], and this variation is partly due to the lack of internationally accepted consensus criteria for the neuropathological diagnosis of VaD. In elderly patients, the prevalence of ‘pure’ VaD ranges from 5 to 78 %. In the oldest-old, that is, ≥90 years, the prevalence of pure VaD drops (to 4.5–46.8 %) but that of mixed AD/VaD increases, reflecting a constant age-related increase of neurodegenerative changes. Rigorous population-based clinico-pathological correlative studies addressing the prevalence of VaD are few, but they are arguably more informative about the actual prevalence of VaD/VCI. In population-based clinico-pathological series, the prevalence of pure VaD ranges from 2.4 to 23.7 %, and that of mixed AD/VaD from 4.1 to 21.6 % [25, 26]. The range is still wide and this may reflect regional differences in managing cardiovascular risk factors and ethnic-related genetic variances. In general terms, these studies show that the prevalence of VaD/VCI is higher in developing countries and Japan. For instance, in a clinico-pathological study from Brazil, where cardiovascular risks are poorly managed, the prevalence of pure VaD was 21.2 %, one of the highest detected in population-based studies [26]. On the other hand, in a retrospective hospital-based study in 1700 consecutive autopsy cases of elderly patients with dementia in Vienna, Austria (mean age 84.3 ± 5.4 years; 90 % over 70 years), pure VaD was observed in 10.7 %, decreasing between age 60 and 90+ from 15.0 to 8.7 % [27]. VaD and VCI are potentially preventable diseases; therefore, studies focusing on its prevalence, incidence and risks factors in the different populations are essential to guide public policies.

## Controversies in clinico-pathological correlation of cerebrovascular disease

At present there are two fundamental issues regarding the assessment and diagnosis of VaD. First, there are no currently accepted neuropathological consensus criteria regarding the assessment of VaD, VCI, cerebrovascular pathology or related lesions [28]. Neuropathological assessment of the post-mortem brain is required to reach a definitive diagnosis and must be carried out in a standardised manner, applying reproducible methods and following generally accepted consensus criteria [29]. Widely used consensus criteria for the pathological diagnosis of common neurodegenerative disease, such as AD and Lewy body disease, have been available for some time [19–21, 30–33]. However, despite several attempts being made without major success [16, 34–36], generally accepted neuropathological criteria for diagnosing VaD are still unavailable. Second, general assumptions regarding the underlying pathology of frequently observed in vivo magnetic resonance imaging (MRI) findings might not always be accurate. Neuroimaging is indeed an important tool in the clinical diagnosis of CVLs and imaging-pathological correlative studies are aiming to bridge the gap between in vivo imaging and post-mortem neuropathology. However, general assumptions regarding the underlying pathogenesis of common in vivo MRI findings are not unequivocally corroborated by neuropathological findings and this may result in inadequate clinical diagnosis and treatment.

## Clinico-pathological correlations and mismatch in vascular dementia and mixed Alzheimer’s disease/ vascular dementia

Various forms of cerebrovascular disorders may lead to cognitive impairment and dementia in the elderly [17]. While pure VaD – most frequently caused by infarctions – is rare, it is generally assumed that cerebrovascular pathology contributes to the development of cognitive impairment in other neurodegenerative diseases, in particular in mixed AD/VaD. Such mixed disorders are frequently observed in the brains of elderly individuals and their prevalence and severity increase with advancing age [37]. In aged individuals, lacunes, microbleeds, WMLs and microinfarcts have been associated with cognitive decline, including reduced mental speed and impaired executive functions [38]. Cerebral SVD may interact with pathophysiological processes in AD either independently of each other or through additive or synergistic effects on cognitive decline [39, 40]. There are several clinical classification criteria for VaD/VCI, such as the NINDS-AIREN criteria, the State of California Disease Diagnostic and Treatment Centers (ADDTC) criteria, the International Classification of Diseases, Tenth Edition ICD-10 criteria and the Diagnostic and Statistical Manual of Mental Disorders, Fifth Edition (DSM-V) criteria. They distinguish between the following: possible VaD – clinical criteria of dementia with focal clinical or imaging signs of one or more infarcts, gait disorder, pseudobulbar palsy, personality and mood changes; probable VaD – all signs of dementia, two or more infarcts followed by dementia and imaging signs of at least one extracerebellar infarct; and proven VaD – clinically proven dementia and pathological demonstration of multiple CVLs and mixed dementia. The diagnosis of VaD/VCI is reflected by recent clinical criteria [41] that are based on evidence of infarcts, white matter hyperintensities (WMH) and microbleeds, using structural MRI. Several autopsy studies have demonstrated that microinfarcts are major risks for VCI; however, microinfarcts can not be detected by 1.5 and 3.0 T MRI or naked eye examination, whereas they may be seen on novel high-resolution 7.0 T MRI [42–45]. However, no accepted and pathologically validated criteria for the diagnosis of VaD/VCI are currently available [46]; therefore, the diagnostic accuracy of possible VaD is still relatively poor, with an average sensitivity of 0.49 (range 0.20–0.89) and an average specificity of 0.88 (range 0.64–0.98) [47, 48]. Cognitive decline has been shown to be weighted on specific pathological lesions in the following ranked order: NFT > Lewy bodies > Aβ plaques > macroscopic infarcts [49]. In neuropathologically defined mixed AD/VaD and SVD, the cognitive impairment profile mirrors that seen in AD cases, that is, all cognitive domains are equally impaired but memory scores are lower than executive scores [50]. This indicates that, regarding the combination of AD and SVD, it is the AD pathology that has the greatest impact on the severity and profile of cognitive impairment. Longitudinal, clinical and neuropathological studies have previously illustrated the impact of AD pathology in mixed AD/VaD, and demonstrate the usefulness of multivariate approaches to understand clinico-pathological profiles, as well as highlighting the current limitations to modelling and predicting cognitive decline and clinical profiles [49]. Nevertheless, the detection of the preclinical stages of cognitive impairment and early AD changes became a reality with the inception of amyloid PET tracers and various Aβ ligands, for example, Pittsburgh Imaging Compound B (PiB), fluorbetapir and flutemetamol [51]. Several studies have illustrated how amyloid PET imaging will improve differentiation between AD and mixed AD/VaD cases of dementia.

Converging evidence suggests that cerebrovascular and AD pathology exert an additive (and/or synergistic) effect on cognitive impairment. Does CVD merely reduce the cognitive threshold needed for overt clinical dementia in AD, or do both factors potentiate AD-specific pathophysiological pathways? Recent neuroimaging studies in cognitively normal elderly people aged 70–90 years suggested that vascular and amyloid pathologies are at least partly independent predictors of cognitive decline in the elderly, and that cognitive reserve seems to offset the deterioration effect of both pathologies on the cognitive trajectories [52].

Concomitant CVLs increase the risk and severity of clinical dementia in elderly individuals meeting the neuropathological criteria for AD [53–55]. However, many studies emphasise additional pathogenesis in older people without dementia, in particular CVLs, with, for example, small or large cerebral infarcts, lacunar infarcts and WMLs reported in 22 to almost 100 % of cases [48, 55–61]. Cerebral infarcts were seen in 21–48 % of seniors without dementia, with a higher frequency of large infarcts [48, 55, 58, 60, 62–64] and CAA [55, 58]. Among 418 participants without dementia in the Religious Order Study (mean age 88.5 ± 5.3 years), 35 % showed macroscopic infarcts; those without macroscopic infarcts had microinfarcts (7.9 %), arteriosclerosis (14.8 %) or both (5.7 %), with only 37.5 % being free of CVLs [63]. In a study of 336 cognitively normal elderly adults, cerebral microinfarcts were seen in 33 % and high-level microinfarcts in 10 % [65]. In another study of 100 elderly participants without dementia (mean age 81.2 ± 5.4 years), CVLs including basal ganglia/deep white matter lacunes were seen in 73 % and CAA in 39 %; only 9 % of these participants were free of CVLs [66]. There were no correlations between CVLs and AD-related pathology in this latter cohort, whereas others reported an inverse relationship between Braak NFT stage and CVLs in autopsy-proven AD [67, 68]. The profile of AD and vascular changes becomes more complex with increased cognitive impairment in older people without dementia and these changes are likely to constitute a major substrate for age-associated cognitive impairment, suggesting a need for rigorous investigation of both neurodegenerative and vascular risk factors in old age [61]. However, the interactions in the pathophysiology between vascular risk factors, CVD and AD pathology, while plausible, are still unresolved.

In contrast to AD, less is known about the impact of CVD in other common neurodegenerative diseases, such as dementia with Lewy bodies (DLB) and frontotemporal lobar degeneration (FTLD). Prevalence reports of CVD in DLB are scarce, but autopsy studies reported a frequency of 20.2–34.4 % [69, 70], which does not differ significantly from controls [70]. In addition, an autopsy study indicated that more advanced Lewy body pathology is less likely to show severe CVD, and therefore suggested that cognitive impairment in DLB appears to be independent of CVD [71]. With regards to the heterogeneous group of FTLD, data in relation to the prevalence and patho-mechanistic role of CVD are very limited and contradictory. One autopsy study reported a frequency of 5.2 % for FTLD-tau and 17.3 % for FTLD-TDP-43 [69]. Some data support a role for SVD in FTLD disease progression [72], while others could not confirm this [69]. Therefore, further studies are necessary to clarify the role of CVD in non-AD neurodegenerative diseases.

In conclusion, the co-occurrence of CVD and AD in the elderly is very frequent [73]. There is evidence suggesting that both lead, in an additive as well as an independent fashion, to cognitive dysfunction. The characteristic pattern of HPτ-related neurodegeneration (i.e. Braak NFT stages) in AD corresponds to a pattern of memory loss that spreads to other cognitive domains. By contrast, the neuropsychological profile associated with VaD shows considerable variation; for example, executive dysfunction often equals or may exceed memory impairment in the SVD-subtype of VaD, but depending on location and severity of CVL all possible types of cognitive impairment may ensue. We anticipate that the availability of comparable measures of AD and VaD pathology from in vivo neuroimaging studies in the future will replace dichotomous classifications of diseases with more sophisticated modelling. However, as of today, the best available models predict less than half of the variance in cognitive performance [49].

## White matter hyperintensities

WMLs histologically encompass structural damage of the cerebral white matter as a result of white matter rarefaction [3]. They are visualised as WMHs on pre- and post-mortem T2-weighted MRI, and they have been associated with a wide range of cognitive deficits [74]. Interestingly, WMHs are frequently seen in individuals both with and without dementia, although WMHs seen in AD are significantly more severe than the ones seen in so-called normal ageing [75–77]. The pathogenesis of WMHs is generally thought to be associated with SVD because vessel wall alterations may lead to chronic hypoperfusion of the surrounding white matter [35]. Although WMHs are currently assumed to reflect SVD, WMHs on T2-weighted MRI are a visualisation of white matter abnormalities and cannot determine the underlying pathogenesis. Previous studies have suggested a multifactorial aetiology of WMHs [78–82] inclusive of SVD-related ischaemia, but also degenerative axonal loss secondary to cortical AD pathology, that is, deposits of HPτ and Aβ. The exact pathological mechanism of degenerative axonal loss is still unclear, but it has been suggested that axonal death occurs simultaneous to grey matter atrophy or via calpain-mediated degradation, activated by AD pathology-related axonal transport dysfunction [83, 84]. Evidence from neuroimaging has shown region-specific white matter changes in patients with AD, most frequently in the posterior deep white matter [75, 85, 86] and corpus callosum [75], which have been directly associated with AD-related cortical atrophy [85, 86].

**Fig. 2:**
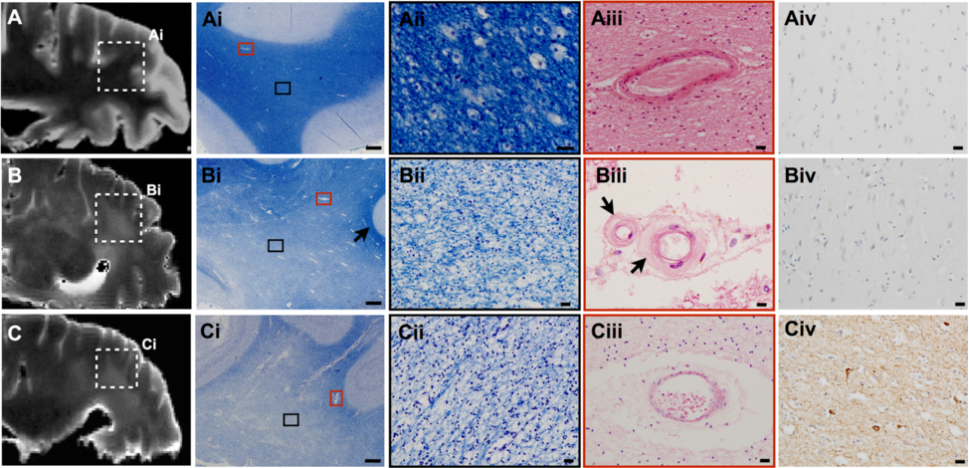
A series of images for three separate cases indicating normal-appearing white matter and the similarity of white matter changes with differing pathogenesis in the deep white matter of the parietal lobe (Brodman area 39/40), as seen on both T2-weighted magnetic resonance imaging (MRI) and on histology. (**A**–**Aiv**) Normal-aged control brain with no obvious white matter changes or small vessel disease (SVD), and no Alzheimer’s disease (AD)-related pathology: (A) post-mortem T2-weighted MRI scan of normal-appearing white matter; (Ai, Aii) corresponding histological magnified image of normal-appearing white matter and a normal white matter artery (Aii); (Aiv) overlying cortex with no hyperphosphorylated tau (HPτ) pathology. (**B**–**Biv**) Normal-aged case that exhibited severe white matter hyperintensities (WMHs)/lesions with SVD but no AD pathology: (B) post-mortem T2-weighted MRI scan indicating confluent WMH; (Bi) corresponding histological magnified image of white matter lesion indicated by widespread pallor of the central white matter with typical sparing of the subcortical U-fibres (*arrow*); (Bii) higher magnification of white matter lesion exhibiting severe rarefaction, that is, myelin and axonal loss; (Biii) white matter arterioles from white matter lesion area exhibiting arteriolosclerosis with hyalinisation (*arrows*) of vessel walls; (Biv) overlying cortex with no HPτ pathology. In this case, one may speculate SVD-related hypoperfusion was the primary cause of white matter changes. (**C**–**Civ**) AD brain exhibiting severe WMHs/lesions and no obvious SVD: (C) post-mortem T2-weighted MRI scan indicating confluent white WMH; (Ci) white matter lesion with severe white matter pallor; (Cii) magnified image of severe white matter rarefaction; (Ciii) white matter arteriole with enlarged perivascular space but no SVD-related fibrosis or hyalinisation; (Civ, overlying parietal cortex exhibiting severe HPτ pathology. In this case, one may speculate white matter changes were the result of degenerative myelin and axonal loss as a result of grey matter atrophy in the overlying cortex or via protease-mediated degradation, activated by AD pathology-related axonal transport dysfunction. MRI scans captured in sagittal plane. Microphotoimages captured from serial sections. Histological stain Luxol fast blue was used for images Ai–ii, Bi–ii and Ci–ii; hematoxylin and eosin stain was used for Aiii, Biii and Ciii. Immunohistochemistry with the AT8 antibody was performed in Aiv, Biv and Civ. Scale bars represent 1000 μm in images A, B and C and 20 μm in images Ai–iii, Bi–iii and Ci–iii

HPτ has been implicated as a principle instigator of degenerative axonal loss in AD. An extensive quantitative neuropathological study revealed that the burden of cortical HPτ in the temporal and parietal lobes was a predictor of WMH severity in AD [87], corroborating previous studies reporting an association between higher Braak NFT stage and increased WMH severity [77, 78, 88], and degenerative axonal loss in temporal [89] and parietal [84] white matter when in proximity to high cortical HPτ pathology burden. Furthermore, the combination of high cerebrospinal fluid (CSF) total-tau and higher parietal WMH volume was shown to predict the clinical conversion from mild cognitive impairment to AD [89],further supporting an association between the two pathologies. Although SVD-related ischaemic damage has long been assumed to be the main factor for the development of WMHs (for review see [90]), neuropathological investigations of patients with AD with severe WMH usually revealed only minimal SVD pathology [84, 89, 91]. However, in cases with minimal neocortical HPτ pathology (Braak NFT stage 0–II), SVD was found to be associated with WMH (Fig. 2) [92].

While theoretically both cortical HPτ pathology and SVD may lead to the development of WMH, it appears that in neurodegenerative diseases such as AD, WMHs are likely to be primarily associated with cortical HPτ pathology. On the other hand, in cases without dementia and in VaD cases, SVD seems to play a role in the development of WMH, which may relate to gliovascular abnormalities and BBB damage [93]. The clarification of the underlying pathogenesis of WMH and respective MRI characteristics is warranted to allow for clear interpretation of white matter neuroimaging and subsequent adequate management of patients.

## Cerebral microbleeds

The term cerebral microbleeds describes the radiological phenomenon of small, well-demarcated, hypointense, round or ovoid lesions detected on T2*-weighted gradient-recalled echo (T2*-GRE) and susceptibility-weighted imaging (SWI) MRI sequences [10]. Microbleeds create a ‘blooming’ effect on T2*-GRE/SWI, but are generally difficult to see on T1-weighted or T2-weighted sequences [10, 92]. Microbleeds have generated interest as a marker of the haemorrhagic consequences of SVD. Microbleeds are common in many different patient populations (healthy elderly, ischaemic stroke, intracerebral haemorrhage [94, 95], AD [96, 97] and VCI [98]). Of note, microbleeds are more prevalent in patients with recurrent stroke than in those with first-ever stroke, and they tend to accumulate over time, indicating a relationship with the progression and severity of cerebrovascular pathology [94]. Microbleeds generate increasingly common clinical dilemmas due to the concern that they may be a marker of future intracerebral bleeding risk [99–104]. In a meta-analysis of 10 prospective studies including 3067 patients with ischaemic stroke or transient ischaemic attack, the presence of microbleeds was associated with a high risk of intracerebral haemorrhage (pooled odds ratio 8.53), raising questions regarding the safety of antithrombotic drugs [105, 106]. Moreover, most available studies suggest that microbleeds are associated with impairment of cognitive function [107, 108], although whether they are directly and independently implicated – or simply reflect more severe SVD – remains uncertain.

Similar to other SVD markers, microbleeds appear to represent a potential link between stroke, brain ageing, dementia and AD [97, 109], but they have not yet resulted in high-quality evidence-based recommendations for stroke and dementia clinical practice nor emerged as a valid surrogate marker for clinical trials in SVD, for example, in intracerebral haemorrhage and VCI. This might be due to the significant gap between the clearly defined markers seen on MRI and their as-yet uncertain pathological basis and pathophysiological mechanisms [109–112]. It is consistently emphasised in the literature that microbleeds are the MRI correlate of extravasation of red blood cells from arterioles and capillaries damaged by a primary haemorrhagic SVD process and, therefore, are potentially strongly associated with haemorrhagic stroke risk. However, microbleeds are also associated with increased subsequent ischaemic stroke risk [113–116], highlighting that they are a marker of a CVD that is simultaneously ischaemic and haemorrhagic, a phenomenon sometimes termed mixed CVD [109, 117]. Nonetheless, histopathological correlation studies suggest that radiologically defined microbleeds generally correlate with focal deposits of blood-breakdown products, predominantly haemosiderin-iron [110, 118]. MRI-histopathological correlation has been underutilised [119, 120], with a total of <70 microbleeds analysed in just a small sample of patients [110–112], often detected using relatively insensitive T2*-GRE sequences at 1.5 T [118]. Technical challenges involved in correlating MRI with histopathology for such small lesions with a widespread distribution in the brain probably account for the small number of brains with microbleeds that have been analysed. Notwithstanding these limitations, when systematic neuropathological examination of SWI-visualised microbleeds is undertaken, the underlying pathologic substrates are actually rather variable, including not only focal accumulations of blood-breakdown products, but also (albeit much less commonly) microaneurysms, small lacunes, vessel wall dissections or (pseudo-) microaneurysms [112, 118, 121, 122].

**Fig. 3:**
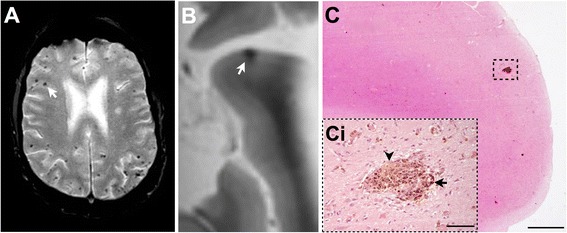
Magnetic resonance imaging (MRI) and histological sections of cerebral tissue exhibiting microhaemorrhages. (**A**) Radiological characteristics of microhaemorrhages inclusive of small, well-demarcated hypointense ovoid lesions (*arrow*). (**B**–**Ci**) Images from an 81-year-old man with dementia and severe cerebral amyloid angiopathy on pathology: (B) post-mortem 7 T MRI scan of hypointense ovoid lesion (*arrow*); (C) magnified image of cortical microhaemorrhage; (*Ci*) increased magnified image of cortical microhaemorrhage – brown deposits are haemosiderin (*arrow*) and yellow deposit is haematoidin (*arrow head*), indicating the microhaemorrhage is subacute. Histological stain hematoxylin and eosin used on images C and Ci. Scale bars represent 1000 μm in image C, and 100 μm in image Ci. Images prepared by Dr S. van Veluw

Although most microbleed pathological correlation studies emphasise blood leakage from nearby damaged small vessels into the brain parenchyma as a mechanism, it must not be assumed that a primary haemorrhagic process fundamentally produces all microbleeds or that the most severely affected vessels are the culprits. Alternative non-haemorrhagic mechanisms for microbleeds, particularly if no tissue damage surrounds the vessel and haemosiderin is limited to the perivascular space, include ischaemia-mediated iron store release by oligodendrocytes [123], phagocytosis of red blood cell microemboli into the perivascular space (termed angiophagy) [121, 124], or even haemorrhagic transformation of small microinfarcts (Fig. 3) [125].

It is widely accepted that, by analogy with spontaneous intracerebral haemorrhage, the pathological processes underlying microbleeds differ according to their location in the brain, with CAA being the most notable correlate of exclusively lobar microbleeds (most often in the occipital and posterior temporo-parietal regions), while ‘hypertensive arteriopathy’ (including a spectrum of neuropathological processes affecting deep perforating vessels such as AS and lipohyalinosis) is strongly associated with predominantly deep microbleeds. The majority of data to date support this hypothesis, but much of the evidence is indirect and largely based on clinical and imaging studies [10, 112, 126–130], rather than extensive direct morphological-pathological analyses [131]. A recent neuropathological study found no direct topographical association between CAA presence or severity and microbleeds (defined only pathologically as haemosiderin-laden macrophages in any brain region) [132]. Whether these microscopic lesions have the same biological significance and underlying mechanisms as radiologically defined microbleeds is not clear [120]. Further exploration of the neuropathological basis of microbleeds will be a key step in clarifying their mechanisms and nature. Along with well-designed observational clinical studies, this greater understanding should allow microbleeds to become useful in clinical management decisions [133]. Until then, the main question of whether a radiologically defined microbleed is always a true microbleed or whether it may also represent haemosiderin deposits, which in turn may or may not stem from a microbleeding event, remains unanswered.

## Additional novel approaches to complement and enhance current post-mortem assessment of cerebral human tissue

With regards to CVL, novel applications of neuroimaging and biochemical methods, as well as additional investigation of neuroinflammation, have been suggested for the assessment of human post-mortem brains. Although these methods are beyond the scope of basic routine diagnostic procedures, the addition of such novel techniques may help to further elucidate the impact of CVD on cognitive performance.

### Post-mortem neuroimaging

Post-mortem MRI provides a technique to complement research, and routine, neuropathological investigations, providing visualisation of cerebral lesions for radiological assessment or a precise location for histological examination. Direct comparison studies have found that gross MRI lesions are almost identical between human in vivo and post-mortem MRI scans [134], with limited effects on MRI characteristics due to the fixation process [135, 136]. A variety of post-mortem MRI approaches have been implemented, including scanning of fixed whole brains or hemispheres [77, 134, 135, 137–140], coronal brain slices [141, 142], un-fixed whole brains [134] and brains in situ [143].

Frequently, post-mortem MRI is used for the detection and assessment of WMH. A recent study investigated the reliability of post-mortem MRI to assess WMH of the deep white matter: 4.7 T MRI scanning was carried out on 40 post-mortem fixed right brain hemispheres, and WMHs in the deep white matter were rated according to the Age-Related White Matter Change Scale (ARWMC) [144] and compared to scores from a thorough histological assessment (based on approximately 1200 sections). The study revealed no significant differences between the post-mortem MRI WMH scores and histological assessments, regardless of the severity of the deep white matter changes, demonstrating that post-mortem MRI is a reliable measure of WMH that can be utilised to complement neuropathological assessment of white matter changes. Of note, routine histological assessment based on five histological sections per brain failed to reliably reflect thorough histological assessment.

Cortical microinfarcts (CMI) are another common lesion found in ageing and dementia, and are considered the ‘invisible lesions’ in clinical–radiological correlation studies [145], visible only upon microscopic examination. Developments in high-resolution 7.0 T MRI have allowed for the detection of CMI in vivo [43]. This approach was utilised and established for the post-mortem detection of several types of CMI by De Reuck and colleagues [45]; fixed coronal slices from 175 demented and non-demented brains underwent a 7.0 T MRI, and mean CMI and cerebral CMI loads were determined and compared to the histological examination, revealing no statistical differences between the two assessments.

Post-mortem MRI has also proved a valuable tool in investigating the pathomechanisms of ischaemic stroke in the human brain. This is of major potential importance because many therapeutic interventions that have proven successful in animal stroke models have not yet been verified in human clinical trials (excluding thrombolysis and hypothermia). Developments in autoradiography of intact human brain sections have allowed for the visualisation of the ischaemic core by creating a ‘potassium map’; a method which identifies the ischaemic core by utilising the disruption of ion homeostasis and subsequent efflux of water. This method allows for the essential targeted tissue sampling of the ischaemic core to facilitate quantitative measurements of tissue components. The method for human brain sections, as described by Csiba and colleagues [146], is reliant upon post-mortem MRI (T1 and T2 weighted) to localise the ischaemic lesions and serve as a gold standard comparison to the potassium map. Of note, in vivo MRI imaging is not appropriate due to the possibility of new focal ischaemic lesions developing. Following post-mortem MRI, the brain is frozen and the region of interest, that is, the brain infarct with the perifocal brain tissue, is cryosectioned using a heavy-duty microtome (LKB 2250 PMV Cryo-microtome; potentionally the entire hemisphere can be cut and examined). The potassium map method can be used to identify the necrotic core, penumbra and perilesional brain on the cryosections [147], with specific samples removed via a micropunch technique [148], allowing for subsequent analysis of water content, proteomics and genetics. Although this combined methodology of post-mortem MRI and potassium mapping is beyond the scope of the routine diagnostic work-up, it is unparalleled in providing targeted tissue sampling for the post-mortem examination of an ischaemic brain in the research setting.

### Biochemical assessment

While clinical, neuroimaging and pathological assessment remain the main approaches for assessing vascular lesions and their association with cognitive impairment and other neurological disturbances, post-mortem biochemistry provides additional insights into vascular function [149] Biochemical assays enable us to measure and investigate the mechanisms of vascular dysfunction, including the activity and level of enzymes and proteins that mediate changes in vascular calibre, permeability and adhesion; cell migration; and vascular maintenance and regeneration. They also allow the measurement of structural protein levels, providing quantitative data on a wide range of vascular and parenchymal cells and extracellular constituents.

**Fig. 4:**
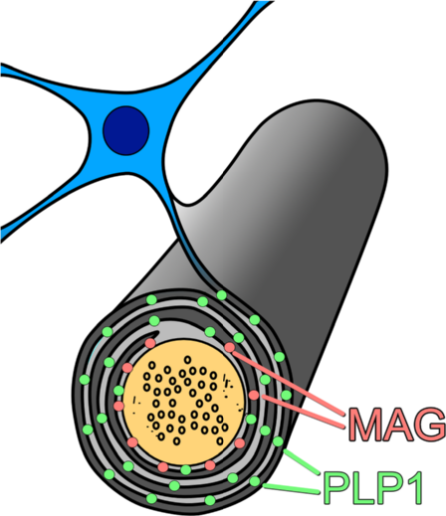
Schematic illustration of the distribution of myelin-associated glycoprotein (*MAG*; *pink dots*) and proteolipid protein 1 (*PLP1*; *green dots*) in the myelin sheath. When the supply of oxygen and glucose is insufficient to meet the metabolic needs of the oligodendrocyte, as occurs in hypoperfusion, the first part of the cell to degenerate is the adaxonal loop of myelin – the part of the oligodendrocyte that is furthest away from the cell body (so-called dying back oligodendrogliopathy). Because MAG is restricted to the adaxonal loop of myelin whereas PLP1 is widely distributed throughout the myelin sheath, hypoperfusion leads to greater loss of MAG than PLP1. In contrast, degeneration of nerve fibres causes loss of both MAG and PLP1. The severity of ante mortem hypoperfusion can be assessed by measuring the ratio of MAG to PLP1. Illustration from [175] with permission from Prof. S. Love

Advantages of including biochemical measurements (in addition to more conventional morphological assessments) include the fact that they are more sensitive for the detection of hypoperfusion, they facilitate more representative sampling (e.g. up to 0.5 ml of tissue in a single homogenate compared with ~5 μl of tissue in a paraffin section) and they yield objective continuous data rather than subjective ordinal scores. Biochemical approaches were recently used to gain some understanding of the pathogenesis of cerebral hypoperfusion in VaD, AD and DLB. Measurement of the levels of myelin proteins with long half-lives but differential susceptibility to hypoperfusion confirmed a significant reduction in the perfusion of the cerebral cortex and white matter in VaD [39, 150]. This was evidenced by a decline in the ratio of myelin-associated glycoprotein (MAG) to proteolipid protein 1 (PLP1). Whereas PLP1 is distributed throughout the myelin sheath, MAG is located in the adaxonal loop of myelin, the first part of the myelin sheath to degenerate when the blood supply is inadequate to meet the energy requirements of the oligodendrocyte (Fig. 4). Biochemical analysis confirmed the significant decline in perfusion of the cerebral cortex in AD as well as VaD [151]. A lower MAG to PLP1 ratio was demonstrable in early AD (Braak NFT stages III and IV) in the precuneus (the first region of the cortex to be affected by a decline in blood flow in AD), indicating that perfusion is inadequate to meet metabolic demand, rather than that hypoperfusion is simply a reflection of reduced metabolic activity [149]. The hypoperfusion in AD could not be attributed to SVD or CAA, with which there was no significant association. However, the severity of hypoperfusion was associated with a marked increase in the concentration of the vasoconstrictor endothelin-1 (EDN1) in the cerebral cortex in AD. A correlation between the level of EDN1 and that of the peptide Aβ42 was also demonstrated, suggesting that it is the accumulation of Aβ42, which upregulates neuronal production of EDN1 by endothelin-converting enzyme-2 [152], that drives the production of EDN1. In contrast, the level of EDN1 did not correlate with that of Aβ40, which upregulates endothelial production of EDN1 by endothelin-converting enzyme-1 [153, 154]).

In the cerebral white matter, the main abnormality associated with hypoperfusion in both VaD and AD has been demonstrated to be non-amyloid SVD [39]. The concentration of EDN-1 in the white matter was found to be reduced in AD, as was that of another vasoconstrictor, angiotensin II, and the activity of angiotensin-converting enzyme, the enzyme responsible for angiotensin II production [149]; these are likely to be adaptive responses to reduced perfusion. However, perfusion of the white matter (as measured by the MAG to PLP1 ratio) has been shown to fall with increasing EDN-1 in the overlying cortex, suggesting that vasoconstriction of perforating arterioles within the cortex probably contributes to hypoperfusion of the underlying white matter in AD.

Additionally, the concentration of von Willebrand factor (VWF) in brain tissue is directly related to the density of microvessels [151, 155]. Measurement of VWF has several advantages over quantitative immunohistochemical methods of assessing microvessel density: the sample size can be much larger (a 0.5 ml homogenate contains **∼** 10^6^-fold greater volume of tissue than a typical paraffin section) and the same homogenate can be used to measure a wide range of related molecules, allowing direct comparison between microvessel density and perfusion, vascular function, and molecules responsible for regulation of vascular growth, tone and permeability. This approach was used to assess possible causes of occipital hypoperfusion in DLB and demonstrated significant reduction in the level of VWF in the occipital cortex (a region known to be hypoperfused in DLB) but not the midfrontal cortex or thalamus [155]. Furthermore, reduction of VWF correlated with a loss of MAG (a marker of hypoperfusion, as noted above), as well as reduced levels of vascular endothelial growth factor (VEGF), which is needed to maintain the vasculature. Finally, reduced VEGF was revealed to be related to the level of α-synuclein, not only in the post-mortem human brain tissue but also in neuronal cell lines engineered to over-express wild-type α-synuclein, suggesting that α-synuclein may down regulate production of VEGF, affecting maintenance of the microvasculature and of cerebral perfusion.

These few examples illustrate the potential of post-mortem biochemical analyses of brain tissue as a means to measure vascular function and to investigate the pathogenesis of vascular dysfunction.

## Neuroinflammation – a contributor to vascular dementia?

**Fig. 5:**
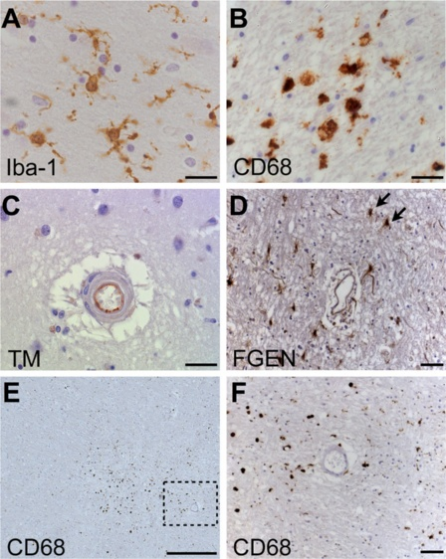
Neuroinflammatory markers in donated human brain tissue from older people. **a** Immunohistochemical labelling for the pan-selective microglial marker Iba-1. **b** Activated microglia in a phagocytic state, with amoeboid morphology, immunoreactive for lysosomal marker CD68 (clone PGM1). **c** Immunoreactivity for endothelial marker thrombomodulin (*TM*) in a small penetrating artery of the anterior putamen. **d** Immunoreactivity for the large plasma protein fibrinogen (*FGEN*) in deep subcortical white matter. Perivascular cells with astrocytic morphology show cellular labelling (*arrows*). **e** A localised cluster of activated microglia (CD68+ (PGM1)), indicating a focal white matter lesion within deep subcortical white matter. **f** Magnified image of E exhibiting a small arterial vessel. Haematoxylin counterstain was used in a–f. Scale bars represent 20 μm in images a, b and c; 100 μm in image e, and 50 μm in images d and f

Aside from the hallmark pathological lesions, there is evidence to suggest a role for immunological and inflammatory mechanisms in the pathophysiology of VaD/VCI. Neuroinflammation encompasses local endothelial activation, leading to the extravasation of fluid (and, sometimes, cells) via a dysfunctional BBB, resulting in oedema and tissue damage in the surrounding parenchyma and eventually leading to the activation of perivascular macrophages, microglia and other glial subtypes (Fig. 5a, b) [156–158].

Clinical studies in patients with symptomatic SVD [159, 160] or WMH [161–163] found elevated levels of circulating biomarkers of endothelial activation, that is, ICAM1, soluble thrombomodulin, interleukin-6 (IL-6) and PAI-1. This suggests that endothelial activation, and a possible inflammatory process, might contribute to SVD and to cognitive decline. A neuropathological study by Giwa and colleagues assessed endothelial activation in small perforating arteries in cases with moderate-severe SVD, and with minimal AD pathology (Braak NFT stage 0–II, and insufficient neuritic plaque pathology to meet CERAD criteria for AD). They found that endothelia were rarely immunoreactive for ICAM1 or IL-6; however, levels of luminal thrombomodulin (depletion of which is a hallmark of activated endothelium) were more pronounced, especially in individual vessels with severe high sclerotic index (Fig. 5c) [164]. The study concluded that local endothelial activation is not a feature of the arteriolosclerosis form of SVD, which is in agreement with evidence from a previous study of brain lysates demonstrating attenuation of inflammatory mediators (MCP-1 and IL-6) in individuals with VaD and mixed dementia, relative to aged control subjects [165]. While BBB dysfunction is often claimed to be part of SVD pathology, neuropathology studies show no conclusive association of BBB markers (fibrinogen, IgG, albumin; Fig. 5d) with SVD. Some neuropathology reports found a positive association between SVD severity and extravascular plasma proteins [166, 167] while others did not [139, 168, 169]. In subcortical white matter, fibrinogen labelling was associated with clinical dementia diagnosis in an AD-free cohort where dementia was likely to be primarily VaD [169]. Observationally, little evidence of leukocyte infiltration has been associated with SVD. Microglia have been shown to be significantly higher in number in the brains of persons with VaD and widespread WMH [79, 170, 171]. Activated microglia (CD68+) are strongly associated with WMLs (Fig. 5e, f) [79, 142].

Elucidation of the role of neuroinflammation in the pathogenesis and pathophysiology of SVD will enable the evaluation of immunotherapies as potential therapeutic options for prevention or treatment of VCI/VaD.

## Conclusion and outlook

It becomes increasingly clear that standardised neuropathological criteria for the assessment of CVD in human post-mortem brains are needed [172]. In order to establish such criteria, Brains for Dementia Research initiated a UK multi-centre collaborative study to formulate evidenced-based Vascular Cognitive Impairment Neuropathology Guidelines (VCING) for post-mortem assessment of CVD of relevance to VCI. Nine neuropathologists undertook a Delphi method series of surveys to agree on a neuropathological sampling protocol and scoring criteria that included assessment of 14 vessel and parenchymal pathologies in 13 brain regions. To validate VCING, the neuropathologists performed blinded assessment of 114 brains from people with little or no AD (Braak NFT stage ≤ III) or Lewy body pathology. Inter-rater reliability analyses showed VCING to be reproducible, with almost perfect agreement among neuropathologists (AC2 coefficient >0.8 [173]) for most scoring, apart from that of AS and microinfarcts, which was more variable (0.4 to ≤0.8). Multivariate logistic regression determined that the best predictive model (area under ROC curve 76 %) of cognitive impairment included moderate/severe occipital leptomeningeal cerebral amyloid angiopathy, moderate/severe arteriolosclerosis in occipital white matter and at least one large infarct (i.e., over 1 cm in diameter). The various combinations of these three pathologies can be used to report a low (<50 %), intermediate (50–80 %) or high (>80 %) likelihood that cerebrovascular disease contributed to cognitive impairment [174].

In addition to the refinement of routine neuropathological scoring criteria, complementary methods such as post-mortem MRI and biochemical assessment are promising tools to investigate CVD. These should be helpful not only to better understand the pathophysiology of VCI/VaD but also to clarify the pathophysiological processes that ultimately lead to characteristic findings of in vivo imaging. The latter seems a timely need, since current assumptions regarding the ‘causes’ of WMH and cerebral microbleeds may not be accurate in all cases and, hence, negatively impact on the diagnostic accuracy of respective clinical diagnoses.

## Abbreviations

AD: Alzheimer’s disease
ARWMC: Age-Related White Matter Change score
AS: Atherosclerosis
Aβ: Amyloid-beta
BBB: Blood–brain barrier
CAA: Cerebral amyloid angiopathy
CERAD: Consortium to Establish a Registry for Alzheimer’s Disease
CMI: Cortical microinfarcts
CSF: Cerebrospinal fluid
CVD: Cerebrovascular disease
CVL: Cerebrovascular lesion
DLB: Dementia with Lewy bodies
DSM-V: Diagnostic and Statistical Manual of Mental Disorders, Fifth Edition
EDN1: Endothelin 1
FTLD: Frontotemporal lobar degeneration
HPτ: Hyperphosphorylated tau
MAG: Myelin-associated glycoprotein
MRI: Magnetic resonance imaging
NFT: Neurofibrillary tangle
NIA–AA: National Institute on Aging–Alzheimer’s Association
PLP1: Proteolipid protein 1
SVD: Cerebral small vessel disease
SVD-AS: Small vessel disease atherosclerosis
SWI: Susceptibility-weighted imaging
VaD: Vascular dementia
VCI: Vascular cognitive impairment
VCING: Vascular Cognitive Impairment Neuropathological Guidelines
VEGF: Vascular endothelial growth factor
VWF: Von Willebrand factor
WMH: White matter hyperintensity
WML: White matter lesion
